# Supplementary material for: Splice-Junction-Based Mapping of Alternative Isoforms in the Human Proteome
Source: Cell Rep. Author manuscript; Available in PMC 2020 Jan 15. (PMC6961840; doi:10.1016/j.celrep.2019.11.026)
Supplement: 2 [file NIHMS1546469-supplement-2.zip › DF1/PXD009737/TestisTrypsin/TestisTrypsin_6_SYNRG_FSTASASHSVPEAGPSLEEK.pdf]

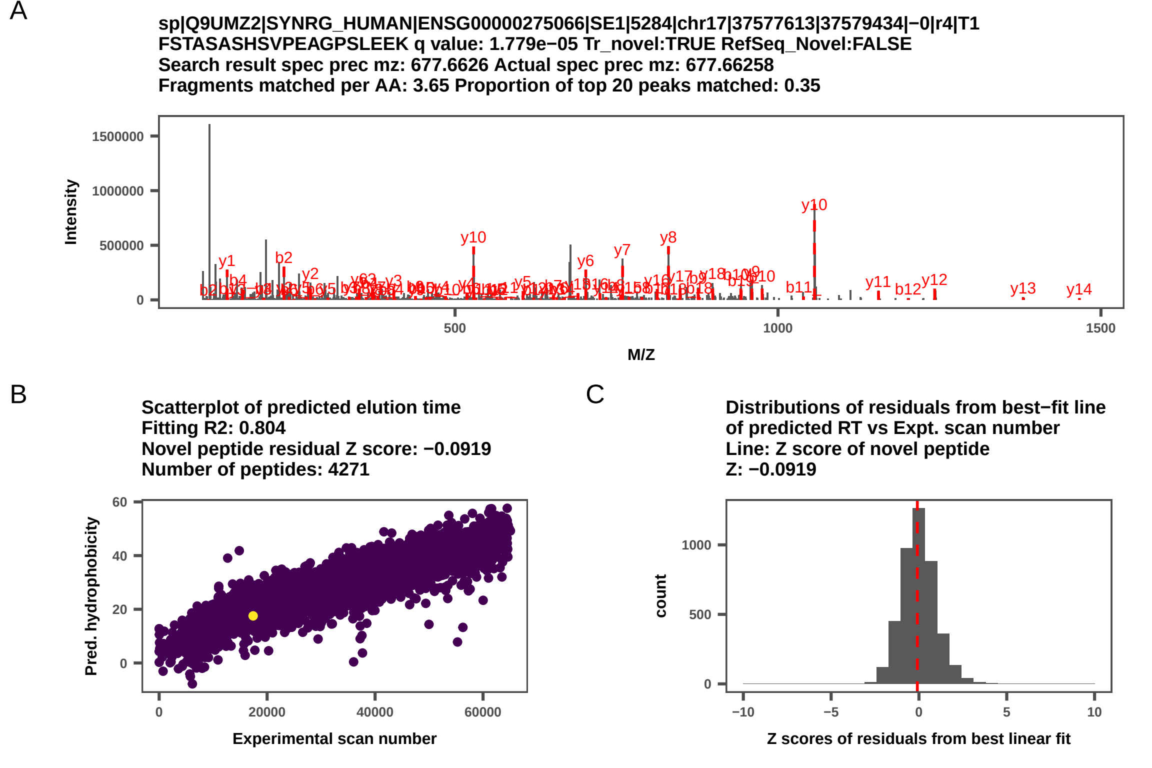
A
sp|Q9UMZ2|SYNRG_HUMAN|ENSG00000275066|SE1|5284|chr17|37577613|37579434|−0|r4|T1
FSTASASHSVPEAGPSLEEK q value: 1.779e−05 Tr_novel:TRUE RefSeq_Novel:FALSE
Search result spec prec mz: 677.6626 Actual spec prec mz: 677.66258
Fragments matched per AA: 3.65 Proportion of top 20 peaks matched: 0.35
Intensity
1500000
1000000
500000
0
500
1000
1500
M/Z
B
Scatterplot of predicted elution time
Fitting R2: 0.804
Novel peptide residual Z score: −0.0919
Number of peptides: 4271
Pred. hydrophobicity
60
40
20
0
0
20000
40000
60000
Experimental scan number
C
Distributions of residuals from best−fit line
of predicted RT vs Expt. scan number
Line: Z score of novel peptide
Z: −0.0919
count
1000
500
0
−10
−5
0
5
10
Z scores of residuals from best linear fit
